# VPS35, the core component of the retromer complex, and Parkinson's disease

**DOI:** 10.1002/ibra.12004

**Published:** 2021-12-09

**Authors:** Ai‐Di Luo, Zu‐Cai Xu, Shu‐Sheng Liao

**Affiliations:** ^1^ Department of Neurology The Affiliated Hospital of Zunyi Medical University Zunyi Guizhou China

**Keywords:** mechanism, Parkinson's disease (PD), retromer complex, vesicle protein sorting 35 (VPS35)

## Abstract

Parkinson's disease (PD) is a neurodegenerative disease that is common in middle‐aged and elderly people, and its onset is related to multiple factors, such as heredity, environment, and age. The vesicle protein sorting 35 (VPS35) gene was found to be a late‐onset autosomal dominant familial PD (PARK17) causative gene. The protein encoded by this gene is located in the endosome and aggregates with other membrane proteins to form a retromer complex, which participates in the membrane protein cycle between the endosome and the Golgi network. Increasing evidence shows that VPS35 may participate in the pathogenesis of PD by affecting autophagy, mitochondria, neurosynaptic transmission, dopamine signaling pathways, and so forth, and it can interact with other disease‐causing genes of familial PD. This article aimed to review the functions of VPS35 and the mechanism of its mutations in PD that have been discovered in recent years.

## Graphical abstract

VPS35 is the core component of the retromer complex; it may play a role in the pathogenesis of Parkinson's disease (PD) by affecting autophagy, mitochondria, neurosynaptic transmission, dopamine signaling pathways, and so forth, and it can interact with other disease‐causing genes (PARKIN, LRRK2) of familial PD.

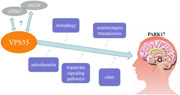

## 1 INTRODUCTION

Parkinson's disease (PD) is a common degenerative disease of the nervous system. It has been reported that the prevalence rate of PD in people older than 60 years of age is approximately 1%, and the prevalence rate in people older than 85 years of age is approximately 4%–5%; in our country, among individuals older than 65 years of age, the prevalence is 1700/100,000, and it increases with age.^1^ However, the pathogenesis of PD is not yet clear and may be related to multiple factors, such as genetic factors, environmental factors, and aging.^2^, ^3^ With the development of molecular genetics, more than 20 disease‐causing genes have been cloned thus far.^4^, ^5^ In 2011, Vilariñno‐Güell et al. used an exome sequencing method to find the c.1858G>A (p.D620N) mutation of vesicle sorting protein 35 (VPS35) in a family with late‐onset autosomal dominant PD in Switzerland for the first time and named the disease subtype PARK17.^6^ In the same year, Zimprich et al. also used this method to discover the c.1858G>A (p.D620N) mutation of VPS35 in an Austrian PD family with an average age of 53 years.^7^ Subsequently, other research groups also obtained similar findings in some individuals and families of PD patients worldwide, suggesting that the VPS35 gene mutation is obviously related to the progression of PD.^8^ It has been found that c. A1858G>(p.D620N) is the most common type of VPS35 mutation. The VPS35 D620N variant in the general population has not yet been determined exactly. At present, the clinical symptoms and neuroimaging of VPS35‐related PD patients suggest that their typical disease spectrum is similar to that of idiopathic PD.^7^ PD patients with VPS35 gene mutations have at least 3/4 of the main motor symptoms of PD clinically, mainly tremor. Subjects occasionally show mild cognitive impairment, and all reported subjects respond to levodopa treatment.^9^, ^10^, ^11^ Studying the function of VPS35 and revealing the pathogenesis of its development in PD could enhance the understanding of DA neuron dysfunction and degeneration in PD and contribute to the genetic diagnosis and treatment of the disease.

### 1.1 Expression distribution, structure, and function of VPS35

The retromer complex can promote the transport and circulation of transmembrane proteins in the endosome–GTN and endosome–plasma membrane. It is usually composed of two major subunits: the cargo recognition trimer (CSC) and the sorting nexin dimer (SNX). The CSC is composed of VPS26, VPS29, and VPS35, and is responsible for identifying and combining goods to be classified.^12^ VPS35 is the core component of the retromer complex, which can regulate the sorting and assembly of transmembrane cargo and direct the transport of specific proteins to the Golgi apparatus or cell surface to play its role.^11^ VPS35 is widely expressed in human tissues, and it is highly expressed in organs and tissues, such as the brain, heart, testis, ovary, small intestine, spleen, skeletal muscle, and placenta.^13^, ^14^ The VPS35 gene is located on chromosome 16q11.2, with a full length of 33 kb, including 17 exons (NM_018206.6), and the encoded protein contains 796 amino acid residues, with a molecular weight of approximately 92 kDa.

## 2 VPS35 PROTEIN AND PD

### 2.1 VPS35 mutation phenotype profile

**Figure 1:**
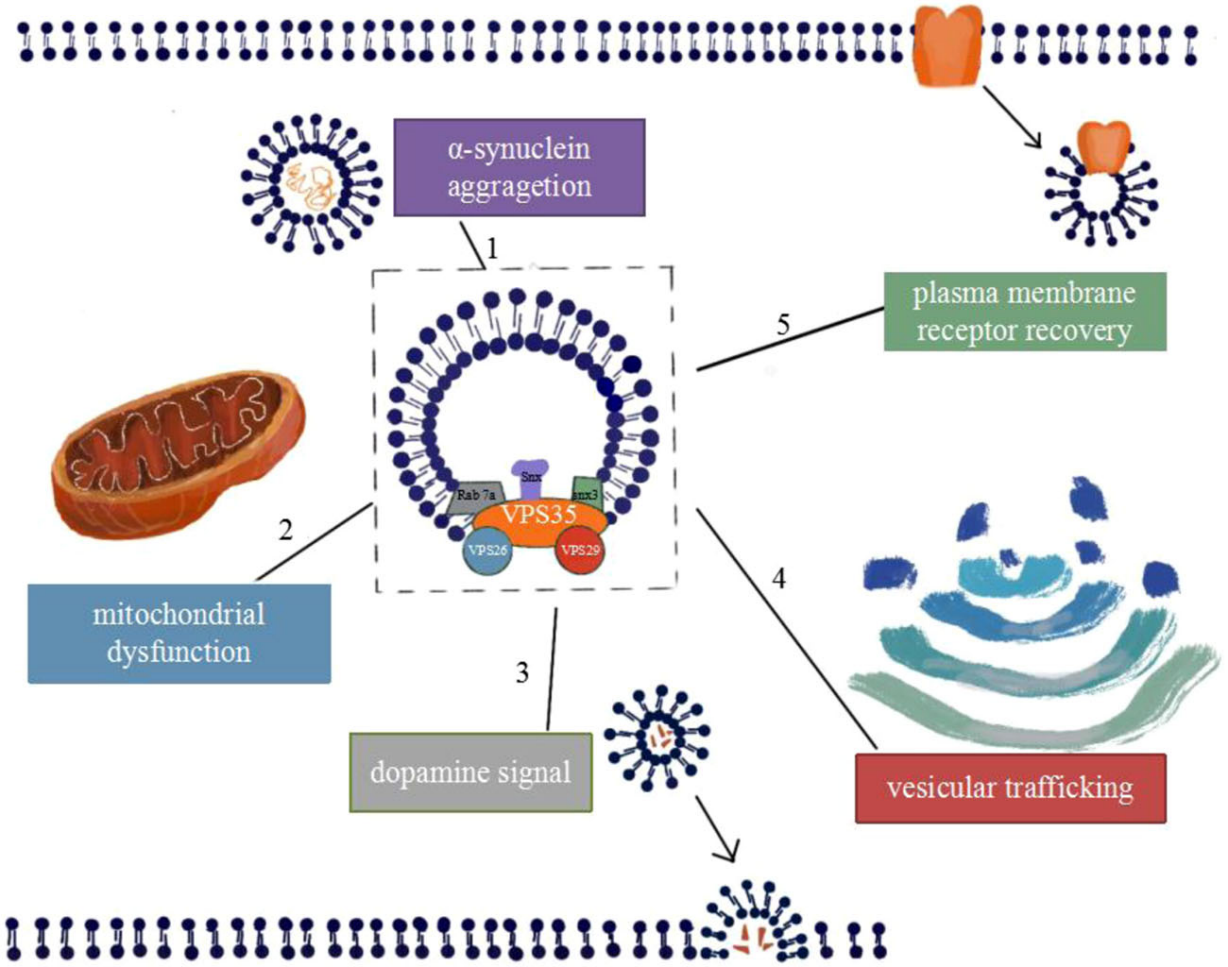
Cellular processes affected by the VPS35 mutation. VPS35, which is the core component of the retromer, along with VPS26 and VPS29, sits at the endosomal membrane and recognizes cargo to be sorted. The retromer, along with retromer‐associated proteins that aid in membrane binding (Snx3 and Rab7a), is responsible for the retrograde transport of several cargo proteins from the endosomal network to either the trans‐Golgi network or the plasma membrane. (1) VPS35 can control α‐synuclein degradation through macrophage autophagy, and endosomal pathways, etc. The VPS35‐d620n mutation and the VPS35 deletion cause α‐synuclein degradation dysfunction; (2) VPS35 indirectly regulates Mfn2 in the mitochondrial membrane. The VPS35 mutation increases the interaction with DLP1, making mitochondrial dynamics prone to excessive division; (2) the VPS35 mutation loses its function of regulating DRD1 transport and signal transduction, and the mutation or defect of VPS35 may interfere with the normal dopamine signaling pathway;  and (4 and 5) VPS35 is the core component of the retromer complex, which can regulate the sorting and assembly of transmembrane cargo and direct the transport of specific proteins to the Golgi apparatus or cell surface to play its role [Color figure can be viewed at wileyonlinelibrary.com]

VPS35 PD has significant clinical and genetic heterogeneity. Different mutation sites, mutation types, and races have different phenotypes. The currently known mutation sites in the VPS35 gene in PD are summarized in Table 1. Also, the cellular processes affected by the VPS35 mutation are detailed in Figure 1.

**Table 1:** Known mutation sites of the VPS35 gene in Parkinson's disease

Type of mutation	Base change	Amino acid change	Phenotype	References
Missense/nonsense	c.96A>C	R32S	PD?	[15]
	c.171G>A	M57I	ADLP?	[7]
	c.723T>G	I241M	ADLP?	[7]
	c.946C>T	P316S	ADLP?	[6]
	c.1463A>G	Q488R	PD17	[16]
	c.1520A>T	Y507F	PD?	[17]
	c.1570C>T	R524W	ADLP	[7]
	c.1576C>T	R526C	PD?	[18]
	c.1679T>C	I560T	LBD?	[19]
	c.1796A>G	H599R	LBD?	[19]
	c.1819A>G	M607V	LBD?	[19]
	c.1858G>A	D620N	ADLP	[6]
	c.1874T>C	L625P	EOAD	[20]
	c.2210C>T	A737V	ADLP?	[21]
	c.2320C>A	L774M	ADLP?	[7]
	c.2359G>A	E787K	PD?	[17]
Splicing	IVS2+33G>A	–	PD?	[22]
	IVS5+79G>A	–	LBD?	[19]
	IVS10−70G>A	–	LBD?	[19]
Small deletion mutation	c.2141 2142delCT	–	–	[23]
Complex rearrangements	Translocation t(15;16) (p11.2;q12.1)	–	–	[24]

Abbreviations: ADLP, autosomal dominant late‐onset Parkinson's disease; EOAD, early‐onset dementia; LBD, Lewy body disease; PD, Parkinson's disease.

## 3 VPS35 AND THE PATHOGENESIS OF PD

### 3.1 VPS35 affects the degradation and aggregation of α‐synuclein

α‐synuclein plays an important role in maintaining synapses and neurotransmitter transport, but the aggregation of α‐synuclein after mutation is a pathological sign of PD. Decreased VP35 levels or VPS35 mutations in the hippocampus of PD mice led to defects in α‐synuclein clearance, leading to the extensive accumulation of aggregates. Additionally, in mouse models, a lack of VPS35 or pathogenic VPS35 mutations can lead to the accumulation and aggregation of α‐synuclein, accompanied by DA neuronal degeneration, decreased DA levels, motor behavior disorders, and lysosomal morphological changes.^25^ In contrast, excessive wild‐type VPS35 expression can inhibit the accumulation of α‐synuclein aggregates and lead to decreased neuron loss and astrocyte proliferation in PD mouse models overexpressing α‐synuclein.^26^ These data indicate that endosomal dysfunction caused by the loss of VPS35 impairs the ability of neurons to cope with the accumulation and removal of α‐synuclein, thereby promoting the progression of PD pathology. In addition, studies have suggested that the lack of retromer complex activity caused by the mutation of the VPS35 gene can cause DWT1 to regulate iron‐ion transmembrane transport pathway obstacles, cause a large amount of iron‐ion deposition, and induce α‐Synuclein aggregation.^27^

Under normal circumstances, α‐synuclein can enter the lysosome through macrophage autophagy, CMA, and endosomal pathways and be degraded by lysosomal hydrolase.^28^ VPS35 indirectly regulates lysosomal activity by sorting receptors for lysosomal hydrolases (such as Sortilin and CI‐MPR). The impaired lysosomal function caused by defects in endosome‐to‐Golgi transport and the incorrect sorting of lysosomal hydrolases (such as CTSD) may promote the accumulation of α‐synuclein in the brain of Drosophila.^29^, ^30^ In addition, the loss of VPS35 in heterozygous KO mice resulted in a decrease in the CMA marker (LAMP2A) because dopamine neurons lacking VPS35 impaired the endosome–Golgi transport of LAMP2A and accelerated the degradation of LAMP2A. Impaired recycling leads to increased lysosomal degradation.^25^ The level of LAMP2A was also significantly reduced in mouse dopaminergic neurons overexpressing the VPS35 D620N mutant, which induced the accumulation of α‐synuclein.^25^ Moreover, the retromer binding to the WASH complex is necessary for autophagy. The VPS35 mutation in a retromer component impairs WASH complex recruitment and the ability of ATG9A to traffic to autophagic compartments.^31^ Therefore, the deletion of VPS35 or the expression of mutant VPS35 D620N had a major impact on lysosomal function and weakened autophagy. These mechanisms are likely to be the cause of neuronal death.

### 3.2 VPS35 and mitochondrial dynamic balance

Mitochondrial dysfunction has always been considered to play an important role in the pathogenesis of familial and idiopathic PD. Mitochondrial fusion protein (MFN2) is a GTPase embedded in the outer mitochondrial membrane that is essential for mitochondrial fusion. Tang et al. found that the absence of VPS35 in neuronal cell lines, DA neurons, and brain tissues reduces the level of MFN2.^32^ The mitochondria of dopaminergic neurons lacking VPS35 are shorter and rounder, suggesting that the loss of VPS35 leads to mitochondrial fragmentation. The expression of wild‐type VPS35 can rescue mitochondrial fusion, while expression of the D620N mutant cannot.^32^ In addition, the interaction of VPS35 and dynein‐like protein 1 (DLP1) can regulate mitochondrial division. The VPS35 mutation increases the interaction with DLP1, enhances the operation of mitochondrial DLP1 through the MDV‐mediated retromer complex, and makes mitochondrial dynamics prone to excessive division, leading to mitochondrial dysfunction and neuron loss.^33^ In short, increasing evidence shows that VPS35 deletion and mutation can affect the dynamic balance of mitochondria, leading to mitochondrial dysfunction.

### 3.3 VPS35 and plasma membrane receptor recovery

Neurotransmitters play an important role in maintaining normal neuronal function. Studies have confirmed that VPS35 deficiency can cause damage to the maturation of mouse dendritic spines and reduce glutamatergic transmission. In VPS35‐deficient neurons, the level of the glutamatergic receptor AMPAR on the plasma membrane surface is reduced.^34^ Another study found that VPS35 is located in dendritic spines and participates in the transport of excitatory AMPAR. The overexpression of VPS35 changes the basic physiological processes of neurons, including excitatory synaptic transmission, AMPAR expression, and synaptic cycling.^35^ In addition, the D620N mutation causes a defect in GluR1 transport, which leads to a defect in downstream synaptic transmission, and neurons lacking VPS35 also show abnormal synaptic transmission and abnormal GluR1 and GluR2 transport.^34^, ^35^ The D620N mutation affects the maturation of dendritic spines and disrupts synaptic transmission, but it is unclear whether these synaptic effects cause neurodegeneration. The glucose transport receptor (GLUT1) is located in the plasma membrane, and the absence of VPS35 in HeLa cells has been shown to cause GLUT1 to be missorted.^36^ However, some studies have shown that the VPS35 D620N mutation does not impair the transport of GLUT1 from the endosome to the plasma membrane.^37^

#### 3.3.1 VPS35 and the dopamine signaling pathway

Dopamine plays a key role in regulating various brain physiological functions by binding to receptors and triggering their endocytosis and signal transduction pathways.^38^, ^39^ The pathological feature of PD is the loss of dopamine neurons in the substantia nigra of the brain. Cataldi et al. reported an independent VPS35 D620N knock‐in mouse model that showed increased dopamine release from the striatum by rapid scanning cyclic voltammetry. They found that VPS35 interacts with dopamine receptor D1 (DRD1), and the overexpression and downregulation of VPS35 increase and decrease the cell surface steady‐state level of DRD1, respectively, and it is accompanied by changes in the phosphorylation levels of the effector molecules of the dopamine signaling pathway, CREB and ERK.^40^ Vanan et al. used a Rosa26‐based transgene expression platform to generate a VPS35 D620N mouse model. The study found that the dopamine level of VPS35 D620N mice was significantly higher than that of nontransgenic control mice.^41^

## 4 INTERACTION BETWEEN VPS35 AND OTHER PD PATHOGENIC PROTEINS

*PARKIN* is an E3 ubiquitin ligase. Mutations in the PARK2 gene encoding *PARKIN* can lead to autosomal recessive early‐onset PD. Drosophila lacking *PARKIN* have a shortened lifespan, decreased climbing ability, and increased sensitivity to paraquat.^42^, ^43^ Malik et al.^43^ crossed VPS35‐deficient fruit flies with VPS35‐mutant transgenic fruit flies, proved that VPS35 overexpression can rescue the defects formed by *PARKIN* mutant phenotype fruit flies, and speculated that VPS35 and *PARKIN* genetically interact. Williams et al.^44^ proved that *PARKIN* interacts with VPS35 and stabilizes ubiquitination in human nerve cells, and the *PARKIN* mutation weakens its ability to ubiquitinate VPS35. In addition, they used adenovirus vectors to express human VPS35 D620N in the substantia nigra of *PARKIN* knockout mice or wild‐type mice, and the overexpression of VPS35 D620N mutants in wild‐type and *PARKIN*‐deficient mice can induce the loss of large numbers of DA neurons.^44^ These results indicate that VPS35 may be downstream of *PARKIN*,^14^ but they did not identify the role of *PARKIN* in mediating the pathogenic effect of the VPS35 mutation, thereby accelerating neurodegeneration.

Leucine‐rich repeat protein kinase‐2 (LRRK2) is a kinase that regulates vesicle trafficking via the phosphorylation of Rab protein subgroups. Its dysfunction may affect the aggregation of α‐synuclein and its pathological changes.^45^ Mutations in the LRRK2 gene can lead to autosomal dominant delayed‐type PD, and G2019S is its most common pathogenic mutation.^46^, ^47^ MacLeod et al. provided evidence to support retromer complex dysfunction in the context of LRRK2‐RAB7L1 pathway defects. In mouse N2A neuroblastoma cells, the expression of LRRK2 G2019S or the knockout of RAB7L1 will cause a significant decrease in the levels of VPS35 and VPS29.^48^ To further study the interaction between VPS35 and LRRK2, Mir et al. discussed the effect of VPS35 on LRRK2‐mediated Rab protein phosphorylation. The knockout of VPS35 resulted in a significant increase in the LRRK2‐mediated phosphorylation of many other Rab proteins.^49^ These data indicate that VPS35 may be located upstream of LRRK2 and play a role in regulating the catalytic activity of LRRK2 kinase. VPS35 controls the activity of LRRK2, and the VPS35 D620N mutation leads to an increase in its function, which may lead to PD through the excessive activation of LRRK2 kinase.^49^

## 5 OUTLOOK

Previous studies on the correlation between VPS35 and PD mainly focused on the clinical manifestations and some possible pathogenesis caused by VPS35 mutations. However, in recent years, research on VPS35 in the field of PD has undergone a qualitative change. As a key protein of the retromer complex, VPS35 participates in a variety of possible mechanisms in the pathogenesis of PD, including affecting autophagy, the dynamic balance of mitochondria, neurosynaptic transmission, dopamine signaling pathway conduction, vesicle transport, other PD pathogenic protein interactions, etc. These findings suggest that increasing the expression level of VPS35 protein in neurons or enhancing its function may be a potential therapeutic target for the treatment of PD. With the rapid development of molecular biology technology, it may be possible to accurately target VPS35 in the future to regulate the clinical symptoms of PD patients.

## CONFLICT OF INTERESTS

The authors declare that there are no conflict of interests.

## ETHICS STATEMENT

The ethics statement is not available.

## AUTHOR CONTRIBUTIONS

Ai‐Di Luo was involved in the main conception of this study and drafted the manuscript; Zu‐Cai Xu was involved in reviewing and editing this paper; and Shu‐Sheng Liao was in charge of acquiring funding, supervising, reviewing, and editing this paper.

## TRANSPARENCY STATEMENT

The authors affirm that this manuscript is an honest, accurate, and transparent account of the study being reported; that no important aspects of the study have been omitted; and that any discrepancies from the study as planned (and, if relevant, registered) have been explained.

## Data Availability

Data sharing is not applicable to this article as no new data were created or analyzed in this study.
